# Streamlining Molecular and Serology Laboratory Operations: A Lean Six Sigma Approach

**DOI:** 10.7759/cureus.80038

**Published:** 2025-03-04

**Authors:** Rashmi Ranjan Guru, Ishani Bora, B Soujanya Kumar, Sumit kumar Sangat, Subhodip Mitra, Vikramjeet Dutta, Rahul Kumar, Swayamprava Dalai, Prasad Bhanap, Meenakshi Sharma

**Affiliations:** 1 Hospital Administration, All India Institute of Medical Sciences, Jodhpur, Jodhpur, IND; 2 Virology, Post Graduate Institute of Medical Education and Research, Chandigarh, Chandigarh, IND; 3 Hospital Administration, Goverment Medical College Ananthapuramu, Ananthapuramu, IND; 4 Hospital Administration, Post Graduate Institute of Medical Education and Research, Chandigarh, Chandigarh, IND; 5 Hospital Administration, All India Institute of Medical Sciences, Kalyani, Kolkata, IND; 6 Microbiology, Safdarjung Hospital, Delhi, IND; 7 Hospital Administration, Symbiosis International (Deemed University), Pune, IND; 8 Biotechnology, Chandigarh University, Mohali, IND; 9 Obstetrics and Gynaecology, Symbiosis International (Deemed University), Pune, IND; 10 Nursing, Post Graduate Institute of Medical Education and Research, Chandigarh, Chandigarh, IND

**Keywords:** lean six sigma, molecular tests, process errors, quality improvement, reducton of errors in the laboratory processes, serology tests

## Abstract

Background

The Lean Six Sigma tools are widely used in various industries, including healthcare, to reduce waste and improve quality. This study investigated how Lean Six Sigma might be used to improve quality control procedures in virology and molecular laboratories. The study intended to minimize errors, expedite laboratory procedures, and shorten turnaround times (TATs).

Methods

A prospective cross-sectional study was conducted at a tertiary care hospital located in India to assess the quality indicators for the processes in a virology laboratory and identify the wastes in those processes. By using the Define, Measure, Analyze, Improve, Control (DMAIC) strategy, the research quantified quality indicators like defects per million opportunities (DPMO) and six-sigma metrics. Subsequently, corrective measures were implemented to reduce the errors. A paired t-test was done by taking the sigma values of pre- and post-intervention phases.

Results

In the pre-analytical phase, a total of 6925 test requisition forms and 12,236 samples were considered, and the sigma value showed drastic improvement after the implementation of the DMAIC strategy. Similarly, in the analytical phase, out of a total of 61 samples, random errors showed an increase in sigma value from 1.71 to 2.47, and reagent contamination showed an increase in sigma value from 1.29 to 2.17. The type of error non-conformity with quality controls showed an increase in sigma value from 1.93 to 2.65, and systemic error showed an increase in sigma value from 2.88 to 3.7. A total of 126 samples were taken in the post-analytical phase, where transcription errors and TAT sigma value drastically increased (transcription error improved from a sigma value of 3.03 to a sigma value of 3.65, and TAT improved from a sigma value of 3.29 to a sigma value of 3.48) by DMAIC strategy. The two-tailed p-value was found to be 0.0269 (p < 0.05), which was statistically significant.

Conclusions

The study highlighted that error reduction directly correlated with a decrease in diagnostic mistakes. Misdiagnoses often lead to alternate treatment methods, causing delays in patient treatment management. Further, the errors reduce the efficiency of the hospital in terms of time and cost. Reducing unnecessary delays in the test process allows clinicians to focus on patient care. The statistical significance of the study showed the effectiveness of the interventions implemented in the study to practical outcomes like reduced misdiagnoses, cost savings, operational improvements, and strengthening the clinical relevance of the study. Further research is needed on these quality improvement steps for the adoptive process flow for the operation process of the laboratories in healthcare setups.

## Introduction

In recent times, the demand for quality healthcare services has been increasing due to factors such as the growing population of patients seeking quality treatment, changing disease patterns, and advancements in medical technology.

The importance of Lean Six Sigma tools has been widely used in various industries, including healthcare, to reduce errors and improve quality. It is often used to standardize the standard operating procedures (SOPs) of clinical practice, focusing on the reduction in the number of errors [1]. This quality tool has already shown excellent results in clinical practice, where the number of errors was negligible. In the context of quality control of serology and molecular tests in a virology laboratory in a tertiary care healthcare center in India, Lean Six Sigma may be applied to streamline the testing process, reduce errors, and improve overall quality [1]. In this context, quality control has become a critical issue, particularly in the field of virology [2].

The virology laboratory is pivotal in diagnosing and treating infectious diseases by utilizing serology and molecular tests. However, these tests encounter quality control challenges, risking result accuracy. Errors in sample handling can lead to false outcomes; therefore, the healthcare centers in India must try to adopt Lean Six Sigma. This methodology, blending Lean's efficiency focus with Six Sigma's quality enhancement, ensures precise and timely test results and is crucial for effective disease management [3]. In virology labs, Lean Six Sigma identifies and rectifies testing process deficiencies, reducing result variation. Implementation improves turnaround time (TAT), patient outcomes, and cost-efficiency, ensuring accurate, timely test results, and is vital for disease management [4]. 

There are few studies done to improve the quality of outcomes of molecular and serology tests in a virology laboratory, specifically by using tools of Lean Six Sigma, and thus, this study was conducted to find out how to use these tools to minimize the errors in the virology laboratory and optimize report-generating time. By removing non-value-adding procedures, the study sought to streamline the laboratory work process and shorten TATs. In accordance with the Define, Measure, Analyze, Improve, Control (DMAIC) strategy, we identified the workflow issues, removed stages that didn't enhance the finished product, enhanced sample flow, and shortened sample transit times. We first used six-sigma metrics and defects per million opportunities (DPMO) to quantify the quality indicators in the pre-analytical phase and then the DMAIC technique to execute corrective actions [5].

## Materials and methods

This was a prospective cross-sectional study conducted at the virology laboratory at an apex healthcare institute, Symbiosis Medical College for Women and Symbiosis University Hospital & Research Centre, Pune, Maharastra, India, providing super-specialty services to patients. Data related to processes in molecular and virology laboratories were included in the study. The study period was six months from April 2023 to September 2023, consisting of pre-intervention, intervention, and post-intervention periods of two months each.

As the study was a quality improvement process, informed consent of the human participants was not required, and the study was approved by the Academic Integrity Committee (AIC) of Symbiosis International University, Pune, India (reference number SMCW/2024/05/100).

Intervention team 

The faculty in charge of the virology laboratory put in the effort for intervention. The laboratory managers were the champions in the outpatient department (OPD), inpatient department (IPD), and emergency room (ER). The phlebotomists, nursing personnel working bedside, the patient care attendants or hospital attendants (who transported the samples), laboratory assistants (who received or entered details of the samples), and laboratory assistants who finally uploaded the reports in the Hospital Information Management System (HIMS) were the members of the intervention team.

Intervention design 

Training Protocols 

Training protocols for the phlebotomist, nurses, hospital attendants, and patient caregivers were formulated and implemented during the intervention phase. 

*Time Investment and Staff Hours* 

The phlebotomists worked 44 hours a week, with an average of 10 minutes per patient for the process of sample collection and packing for the transportation to the laboratory. The nurses worked 40-48 hours a week with an average of 30 minutes per patient for all work related to blood sample collections, drug administration, and assisting the undergoing procedures. 

Training Materials

The training of phlebotomists and healthcare staff took eight hours on a single day, including both classroom and hands-on training. The training materials included techniques for searching the vein, techniques for blood drawing, and other samples. For improving patient communication skills, classes were taken on organizational behavior (OB) by the faculty of the department of hospital administration. Teamwork and leadership management training were conducted for the nurses and the doctors handling incidents during the whole process from blood collection to reporting.

*Supervision Methods* 

The supervision methods comprised initial supervision with ongoing supervision by regular check-ins and mentorship, followed by monitoring and feedback processes by the supervisors and team leaders. A continuing educational program with brief refresher sessions at regular intervals was done throughout the intervention. The control phase in the DMAIC methodologies was important for the improvements, particularly in implementing the systems and processes that will continue monitoring and ensure the long-term effectiveness of the interventions. 

For post-intervention sustainability, standardizing best practices of standard operating procedures (SOPs) accessible to all staff consisted of steps of intervention, guidelines for the staff to carry out their roles effectively, and quality safety protocols. Performance monitoring and measurement by use of key performance indicators (KPIs) was conducted in the form of digital data collection and monitoring by a dashboard. Regular audits and reviews were done by the audit process, and root cause analysis for deviations and necessary modifications was implemented from time to time. A robust feedback mechanism technology and continuous improvement on lessons learned were considered for the study. 

Quality indicators

The quality indicators to detect errors in the processing of samples included in the study were insufficient sample volume, inappropriate sample transportation and storage methods, hemolyzed samples, lipemic samples, collection in the wrong vacutainer, mislabeled samples, samples lost or not received, sampling errors, receiving time, non-conformity with controls, random errors, calibration shift, reagent contamination, systematic error, transcription errors, and TAT. In pre-intervention and post-intervention phases, quality indicators like age, gender, date of collection of samples, unique hospital identification number of the patient (UHID), diagnosis, type of specimen, details of treating physician with signature, and errors concerning the test inputs were used for filling out the test requisition forms.

Quality tool

The quality tool used for this study was the DPMO (*https://www.sixsigmaonline.org/*) in the steps of the laboratory process. From the DPMO value, the sigma value was calculated.

The DPMO was calculated by the following formula:

 DPMO = $\frac{Number~of ~Defects}{(Number~ of~ Units\times Oppurtunities~ per~ Unit)}\times10,00,000$

After getting the DPMO value, the corresponding *sigma value* was found by using a sigma metrics table [6].

Data collection

From April 2023 to September 2023, the laboratory received a total of 12,236 samples. Data were collected from the registers, process champions, the intervention team, and the laboratory information management system that is part of the HIMS. The study included molecular and serological assays, whereas other virology laboratory procedures were excluded. Data were acquired using manual records and entered into a Microsoft Excel sheet (Microsoft Corporation, Redmond, Washington, United States).

Delays in the reception area were measured at regular intervals throughout the week, and an average of the delayed time was calculated. The intervention team worked on potential sources of errors such as sample sorting, allocation (sample handling), and sample labeling (barcoding) on the sample tubes. Types of errors, probable causes of errors, and interventions are shown in Table 1.

**Table 1:** Pre-analytical, analytical, and post-analytical types of errors, probable causes of errors, and interventions

Error types	Probable causes of errors	Interventions
Pre-analytical phase		
Hemolyzed sample	Incorrect phlebotomy technique and transportation of samples	Regular training of staff on phlebotomy techniques. Centrifugation with accurate time. Transportation methods & temperature maintenance.
Insufficient sample	Lack of awareness regarding the volume of samples required in pediatric patients	Written guidelines on sample volume were needed as references. Trainings were done at regular intervals.
Lipemic sample	Collection under a non-fasting state (Hyperlipidaemia)	Training of phlebotomists to update knowledge at the time of sample collection. A written chart on ‘the time of sample collection' was used as a reference
Incorrect identification of patient	Lack of knowledge/lax attitude of the ward staff	Training of the staff. Format for making the correct sticker/barcodes of the patient
Requisition slip without sample	Lack of knowledge/lax attitude of the phlebotomists/staff involved in sample collection and test ordering	Training of phlebotomists with checklists
Illegible handwriting	Lack of knowledge/lax attitude of doctors & staff.	Training of the junior doctors & staff at periodic intervals.
Wrong vials	Lack of knowledge/lax attitude of the phlebotomists/staff involved in sample collection.	Training of the staff. The barcodes were applied to the vacutainer before collecting the sample.
Analytical phase		
Non-conformance with quality control	Old quality control methods, improper storage processes	Written-ready references were given for the appropriate storage of samples in all analysis steps
Random error	Unknown cause	The team leader of the intervention team was contacted when random errors arose for an immediate solution.
Calibration drift	Unstable reagent, refilling reagent, calibration data.	Schedule of calibration made & put on the machine. Reagent details were printed & put on the machine for ready reference.
Reagent contamination	Reagent mix-up, improper storage	Standard Operating Procedure (SOP) for the stepwise reagent used was printed & put in the workplace.
Errors related to Equipment	Probe, lamp, blocked tubing Inherent technical problems/routine wear and tear	Technical errors, so the technician/supervisor was allotted to the intervention team to be contacted, and the on-call contact number was displayed in the laboratory.
Post-analytical phase		
Errors in transcription	Human error while copying data onto the request slips	Reports supervised by the junior doctor on duty before dispatch.
Prolonged turnaround time	Issues about irregular electricity, water shortage, and instrument failure	Engineering & biomedical issues were immediately sent to the hospital administration for prompt action.

Data analysis

In the post-intervention phase, DPMO and sigma metrics were recalculated, followed by DMAIC approach implementation and staff training. The delays in timings of the receiving of samples were recorded on a daily basis, and an average delay time was calculated by using IBM SPSS Statistics for Windows, Version 20.0 (2011; IBM Corp., Armonk, New York, United States). Sample handling procedures and re-barcoding incidents were evaluated for potential dangers and mistakes. The data were tabulated with Microsoft Excel 2010 and were imported to IBM SPSS Statistics for Windows, Version 20.0, for statistical analysis by conducting paired t-tests with p-values <0.05, which proved to be statistically significant.

## Results

Errors in the laboratory

**Figure 1:**
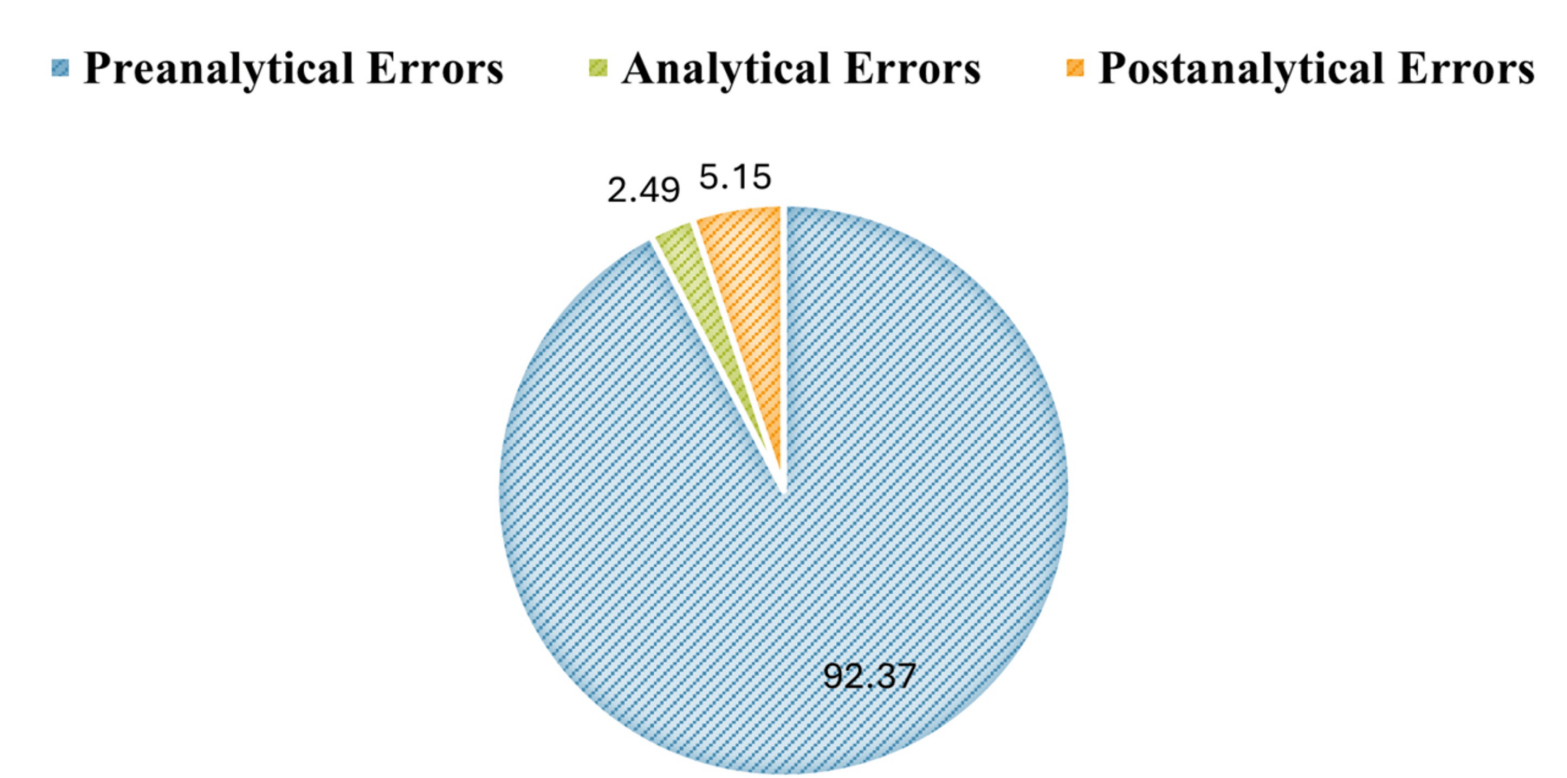
Percentage distribution of errors in pre-analytical, analytical and post analytical phases in piechart

During the study period from April 2023 to September 2023, the laboratory received a total of 12,236 samples. Of these 12,236 samples received, 2449 were found to be with errors. The errors in different phases were found to be 2262 (92.37%) in pre-analytical, 61 (2.49%) in analytical, and 126 (5.15%) in the post-analytical phases of the process. The highest errors were observed in the pre-analytical phase, with a total of 2262 out of 12,236 samples collected. The percentage distribution of errors in the laboratory process is shown in Figure 1.

In the pre-analytical phase, in the test requisition forms, the diagnosis was not mentioned in 66.04% (n = 4573) of the forms, followed by the absence of details of the treating physician in 60.84% (n=4213) of the forms. On analyzing sample collection errors, delay in receiving time constituted 10.67% (n=1303), followed by the collection of samples in the wrong container (3.1%; n=379).

In the analytical phase, a total of 61 errors were found (2.49% of the total errors). Reagent contamination accounted for 40.9% (n=25) of the most common analytical issues found, followed by 31% (n=19)of the random errors. Additional sources of analytical mistakes included nonconformity with 15 events in quality control, one event in systematic error, and two events in calibration shift. In the post-analysis phase, over the course of six months, 126 inaccuracies were noticed; 12 (9%) cases of transcribing problems were noted and delayed TAT contributed 91% (n=114) of errors.

In the pre-analytical phase, a total of 6925 test requisition forms and 12,236 samples were considered, and the sigma value showed drastic improvement after the implementation of the DMAIC strategy. Similarly, in the analytical phase, out of a total of 61 samples, random errors showed an increase in sigma value from 1.71 to 2.47, and reagent contamination showed an increase in sigma value from 1.29 to 2.17. The type of error non-conformity with quality controls showed an increase in sigma value from 1.93 to 2.65, and systemic error showed an increase in sigma value from 2.88 to 3.7. A total of 126 samples were taken in the post-analytical phase, where transcription errors and TAT sigma value drastically increased (transcription error improved from a sigma value of 3.03 to a sigma value of 3.65, and TAT improved from a sigma value of 3.29 to a sigma value of 3.48) by DMAIC strategy. The two-tailed p-value was found to be 0.0269 (p < 0.05), which was statistically significant. 

The sigma values in different phases of intervention are given in Table 2.

**Table 2:** Results of the sigma value comparison among pre-intervention, intervention, and post-intervention phases As the study is observational, the chances of missing data were the least.

Error category in different phases	Sub-category	Error type	Sigma level (pre-intervention) (April 24 – May 24)	Sigma Level (Intervention) (June 24 – July 24)	Sigma Level (Post-Intervention) (August 24-September 24)
Pre-Analytical (n= 19161)	Test requisition forms (n=6925)	Age	3.9	4.01	4.2
		Gender	3.9	4.02	4.24
		Date of collection	4.02	4.22	4.51
		Identification numbers	4.39	4.39	4.63
		Diagnosis not mentioned in requisition forms	1.66	2.02	2.17
		Type of specimen not mentioned	3.06	3.19	3.7
		Physician details and signatures missed	1.74	2.14	2.19
		Errors concerning test input	4.39	4.42	4.63
	Samples (n=12236)	Insufficient sample volume	3.8	3.92	3.97
		Inappropriate sample transportation	4.38	4.6	4.73
		Haemolyzed sample	3.91	3.93	4.04
		Lipemic sample	4.04	4.24	4.6
		Collection in the wrong vacutainer	3.62	3.64	3.72
		Mislabelled samples	3.66	3.66	3.75
		Samples lost/not received	3.77	3.87	4
		Wrong sample	4.16	4.24	4.45
		Receiving time	2.91	3.12	3.4
Analytical (n=61)		Non-conformity with quality controls	1.93	2.31	2.65
		Random errors	1.71	2.31	2.47
		Calibration shift	1.0	1.0	2
		Reagent contamination	1.29	1.93	2.17
		Systemic error	2.88	2.88	3.7
Post-Analytical (n=126)		Transcription errors	3.03	3.36	3.65
		Turn-around time (TAT)	3.29	3.38	3.48

## Discussion

The test report of blood samples is the most essential requirement for the diagnosis of most diseases and the planning of further treatment. In the whole process of sample testing with three phases: pre-analytical phase, analytical phase, and post-analytical phase, the analytical phase of the testing process was found to be a major area of concern in the serology and molecular biology laboratory, similar to other studies [7,8].

The pre-analytical phase errors constitute more than 60% of the errors, which was also seen in most studies [9]. Other studies showed that in the pre-analytical phase of tests, incomplete or wrong filling of the requisition forms, inadequate training of staff, and improper collection procedures were three main areas of concern [10,11]. Among the pre-analytical errors, incomplete details of the physician (60.84%), missing diagnosis details, and an incompletely filled history of the patient were given priority to reduce the errors, thereby increasing the sigma value. All the details were filled in the test requisition form (TRF) by the doctors and the staff of the hospital. Training and written ready reference slips improved the process [12]. Other studies showed that the pre-analytical phase had errors during the process of collecting samples, which were mainly hemolyzed, clotted, insufficient, or incorrect [13,14]. Similar results were found in this study. A study on pre-analytical errors emphasized that monitoring quality indicators improved the process [15], which was also found in this study. Another study emphasized that monitoring the process to reduce errors in the steps was successful with constant supervision of the quality indicators [16].

The analytical phase was the most important step of the whole process, where the samples of the whole process were to be analyzed and the correctness of the results depended on this stage. In the analytical phase of the process, reagent contamination (2.49%) was the most common error in the current study. The adoption of close interdepartmental cooperation is important in the implementation of quality control methods; this is also recommended by a study by Goswami et al. [17]. Abnormal patient results due to calibration drift were the reason for repeat calibrations. The calibration of a parameter was considered to be within limits if the optical density (OD) of the reagent and the factor generated following the calibration procedure fell within the range specified by the manufacturer. Quality control material was reconstituted due to abnormal results. This can be attributed to inappropriate storage of quality control material by the laboratory staff [18].

The post-analytical phase included quality indicators like transcription errors and TAT. The post-analytical phase constituted 5.15% of the total errors. This study showed that transcription errors comprised 9% of the post-analytical errors. Continuous and periodic training and the use of ready reference decreased the errors in the transcription, which was also mentioned in another study [19]. The TAT was reduced below the prescribed TAT by training of the staff and by the use of ready references [20,21]. 

**Figure 2:**
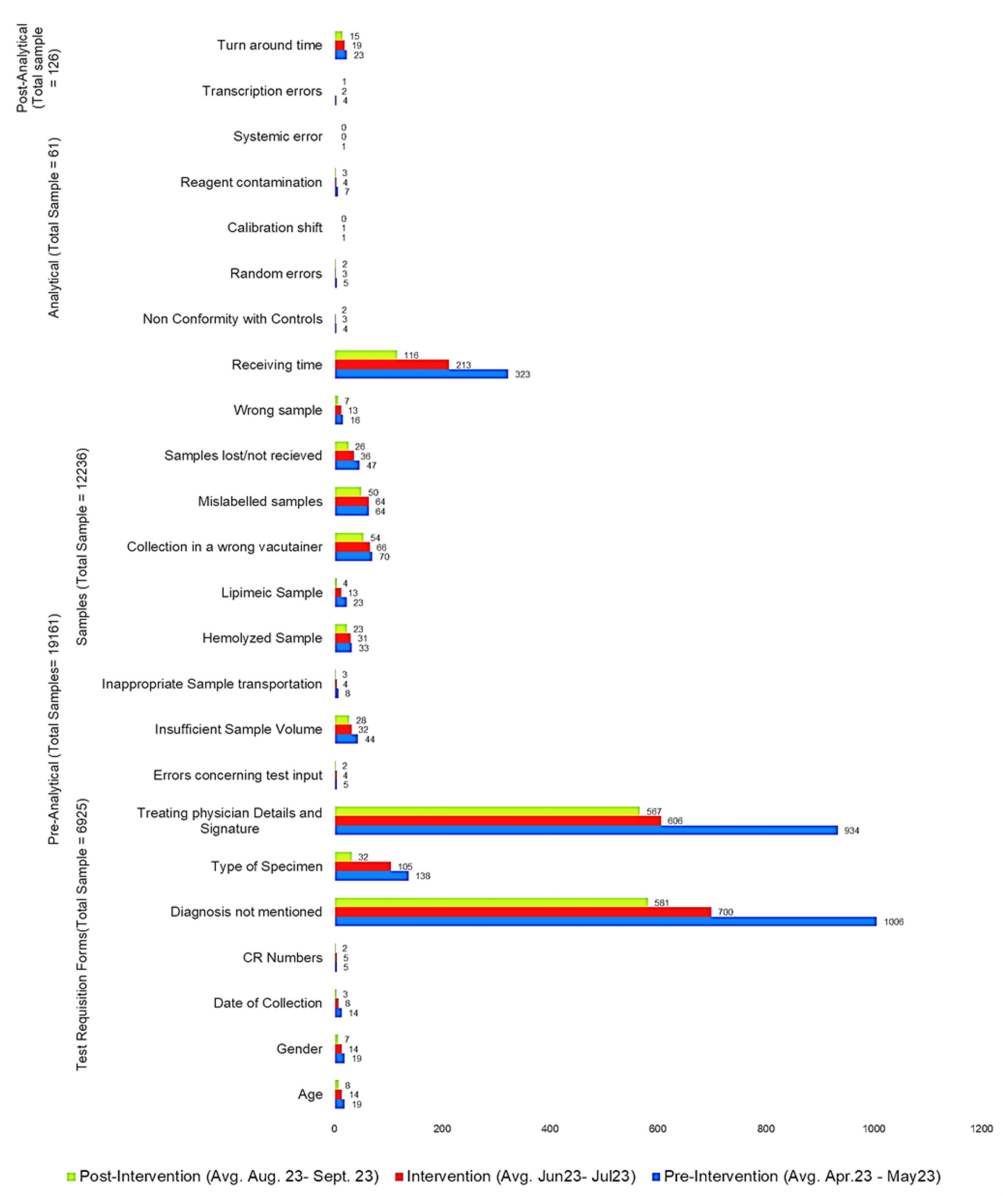
The impact of Lean Six Sigma interventions causing decrease in frequency of errors

The impact of Lean Six Sigma interventions causing a decrease in the frequency of occurrence of errors is shown in Figure 2. 

The study utilized interventions for reducing the errors in each step of the samples, starting from the collection of samples to the dispatch of reports from the laboratory. The interventions elevated the efficiency of the entire process in the virology laboratory by reducing errors and delays in the processes, which resulted in enhanced patient outcomes and overall patient satisfaction. The statistical significance has a positive correlation with the practical significance in the improvement of the performance of the process. 

Recommendations

In this study, a sigma value less than 4.0 was considered for the limit to do corrective and preventive action, increasing the efficiency of the process. A sigma value of 1.1 obtained in the pre-analytical phase was considered poor performance. Hence, the diagnosis of the patient in the test requisition forms and training for its continuation were important. As per the World Health Organization (WHO) standards, a minimum of two identities were needed for the identification of samples; the Unique Health Identification Number (UHID), age, and sex are the best identifiers for the patient’s sample [22]. To improve the sigma value in TAT, focus should be given to process improvement and performance monitoring.

Limitations

The study's potential limitations include a lack of proper quality control materials or SOPs, inadequate software for the Laboratory Information Management System (LIMS) data analysis, operator bias, and staff interest in quality improvement steps. Some data were collected verbally from the staff members, which could have limitations. The potential resistance to change was the staff behavior and wrongly perceived work culture. The virology laboratory showed a drastic change in the process of the operation in comparison to other laboratories, despite the work being like a super-specialty department. 

## Conclusions

Complete quality control encompasses all phases of sample handling, from ordering tests to the physicians' ultimate assessment of the results. Laboratory quality standards can be significantly impacted by the identification, assessment, and development of remedial methods. To ensure patient safety and quality throughout the whole testing process, it is crucial to identify quality indicators at every stage of the process. Quality indicators are an effective technique for determining the likelihood of mistakes during the pre-analytical phase procedures. Promoting optimal phlebotomy techniques, training, and sample transportation protocols was found to be crucial in smoothing the operation of a laboratory. Laboratories working in microbiology, immunology, and genetics can benefit from adopting similar techniques for sample collection until the results are reported. By identifying deficiencies and minimizing errors, healthcare centers can better meet the demands of an evolving healthcare landscape. 

The study highlighted that error reduction directly correlated with a decrease in diagnostic mistakes. Misdiagnoses often lead to alternate treatment methods, causing delays in patient treatment management. Further, the errors reduce the efficiency of the hospital in terms of time and cost. Reducing unnecessary delays in the test process allows clinicians to focus on patient care. The statistical significance of the study showed the effectiveness of the interventions implemented in the study to practical outcomes like reduced misdiagnoses, cost savings, operational improvements, and strengthening the clinical relevance of the study. Further research is needed on these quality improvement steps for the adoptive process flow for the operation process of the laboratories in healthcare setups. 

## References

[1] Rathi R, Vakharia A, Shadab M (2022). Lean six sigma in the healthcare sector: a systematic literature review. Mater Today Proc.

[2] McDermott O, Antony J, Bhat S (2022). Lean Six Sigma in healthcare: a systematic literature review on motivations and benefits. Processes.

[3] Thakur V, Akerele OA, Randell E (2023). Lean and Six Sigma as continuous quality improvement frameworks in the clinical diagnostic laboratory. Crit Rev Clin Lab Sci.

[4] Inal TC, Goruroglu Ozturk O, Kibar F, Cetiner S, Matyar S, Daglioglu G, Yaman A (2018). Lean six sigma methodologies improve clinical laboratory efficiency and reduce turnaround times. J Clin Lab Anal.

[5] Jain P, Chauhan P, Uppal V, Debnath E, Gogoi S, Chatterjee P (2022). Quantification of pre-analytical quality indicators in a clinical laboratory and formulating the Lean Six Sigma DMAIC strategy. J Indian Med Assoc.

[6] (2025). SlideServe: Sigma level conversion table. https://www.slideserve.com/marc/sigma-level-conversion-table.

[7] Westgard JO, Darcy T (2004). The truth about quality: medical usefulness and analytical reliability of laboratory tests. Clin Chim Acta.

[8] Regan M, Forsman R (2006). The impact of the laboratory on disease management. Dis Manag.

[9] Nevalainen D, Berte L, Kraft C, Leigh E, Picaso L, Morgan T (2000). Evaluating laboratory performance on quality indicators with the Six Sigma scale. Arch Pathol Lab Med.

[10] Wiwanitkit V (2001). Types and frequency of preanalytical mistakes in the first Thai ISO 9002:1994 certified clinical laboratory, a 6 - month monitoring. BMC Clin Pathol.

[11] Plebani M, Ceriotti F, Messeri G, Ottomano C, Pansini N, Bonini P (2006). Laboratory network of excellence: enhancing patient safety and service effectiveness. Clin Chem Lab Med.

[12] Kulkarni S, Ramesh R, Srinivasan AR, Silvia CR (2018). Evaluation of preanalytical quality indicators by Six Sigma and Pareto's principle. Indian J Clin Biochem.

[13] Goswami B, Singh B, Chawla R, Gupta VK, Mallika V (2010). Turn around time (TAT) as a benchmark of laboratory performance. Indian J Clin Biochem.

[14] Chhillar N, Khurana S, Agarwal R, Singh NK (2011). Effect of pre-analytical errors on quality of laboratory medicine at a neuropsychiatry institute in north India. Indian J Clin Biochem.

[15] Gajjar DM, Patel DA, Jain DS (2016). Monitoring of quality indicators in pre analytical phase of testing in the clinical biochemistry laboratory of a tertiary care hospital attached with Government Medical College. IOSR J Dent Med Sci.

[16] Khalifa M, Khalid P (2014). Improving laboratory results turnaround time by reducing pre-analytical phase. Integrating Information Technology and Management for Quality of Care.

[17] Goswami B, Singh B, Chawla R, Mallika V (2010). Evaluation of errors in a clinical laboratory: a one-year experience. Clin Chem Lab Med.

[18] Mira NO, del Remedio Guna Serrano M, Cardona CG, Pérez JL (2008). Quality control in molecular microbiology [Article in Spanish]. Enferm Infecc Microbiol Clin.

[19] Lokesh K, Samanta AK, Varaprasad G (2020). Reducing the turnaround time of laboratory samples by using Lean Six Sigma methodology in a tertiary-care hospital in India. 2020 International Conference on System, Computation, Automation and Networking (ICSCAN).

[20] Wallace P, McCulloch E (2021). Quality assurance in the clinical virology laboratory. Encyclopedia of Virology.

[21] Pati HP, Singh G (2014). Turnaround time (TAT): difference in concept for laboratory and clinician. Indian J Hematol Blood Transfus.

[22] (2007). Patient Identification.

